# Supplementary material for: Environmental Selection Shapes Bacterial Community Composition in Traditionally Fermented Maize-Based Foods from Benin, Tanzania and Zambia
Source: Microorganisms. 2022 Jul 5;10(7):1354. doi: 10.3390/microorganisms10071354 (PMC9318576; doi:10.3390/microorganisms10071354)
Supplement: Supplementary file 1 [file microorganisms-10-01354-s001.zip › microorganisms-1750922-supplementary.pdf]

Supplementary Materials

SX Read Quality

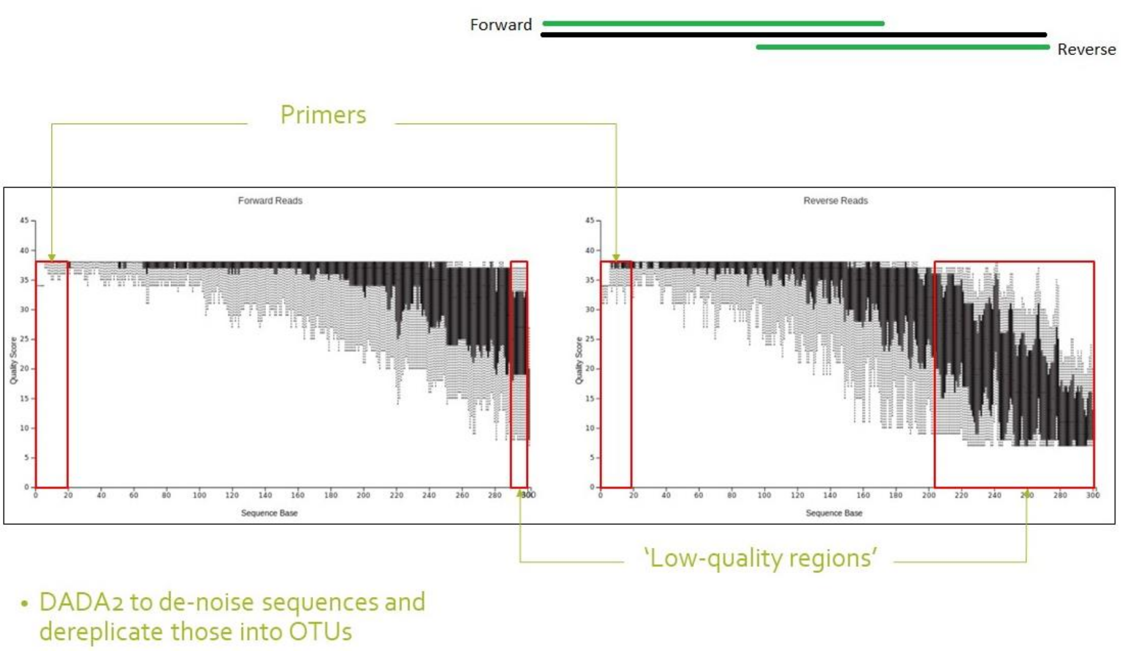

Figure S1. Read quality visualization using demux**.** The left plot represents all forward reads and the right plot all reverse reads. Sequence indices are on the x-axis and corresponding quality on the y-axis. The first 20 basepairs correspond to the primers. The lowest quality regions were on the end of the sequences. Primers and low-quality regions were removed using DADA2.

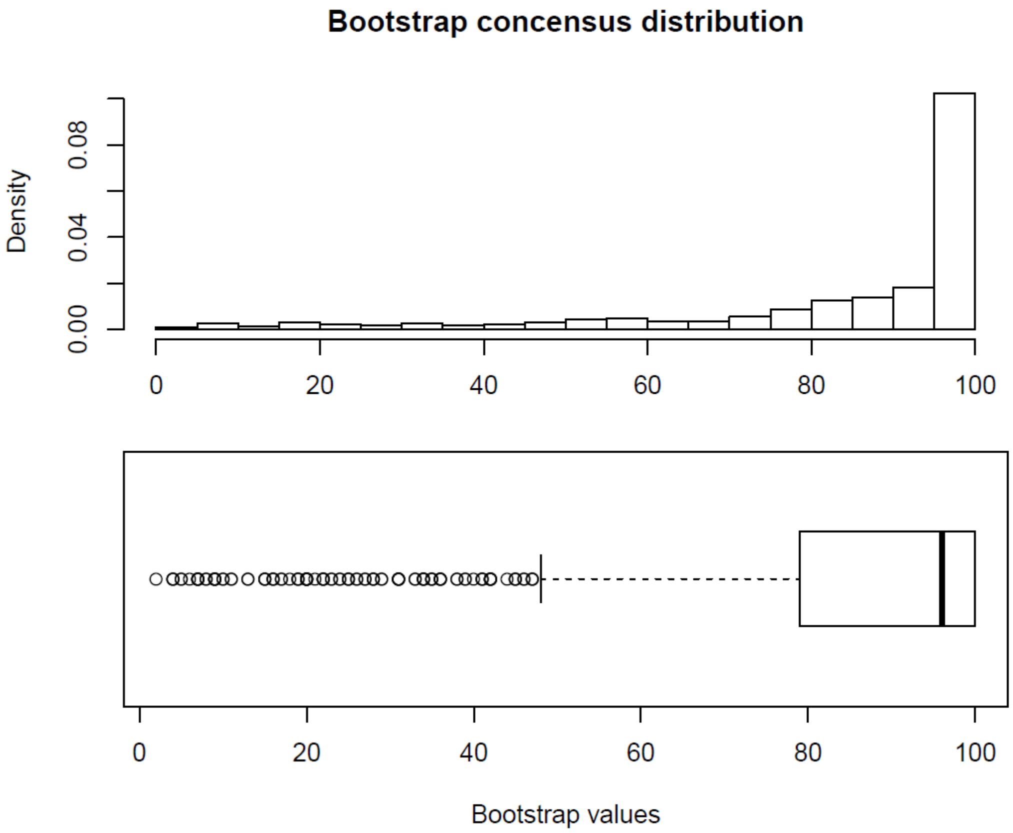

Figure S2. Original bootstrap values distribution phylogenetic tree. The 2000 bootstrap replicates of the IQ-Tree phylogenetic trees ranged between 100 and 5% consensus, most observations being above 80%. Splits with bootstrap values below 50% were removed by merging those splits.

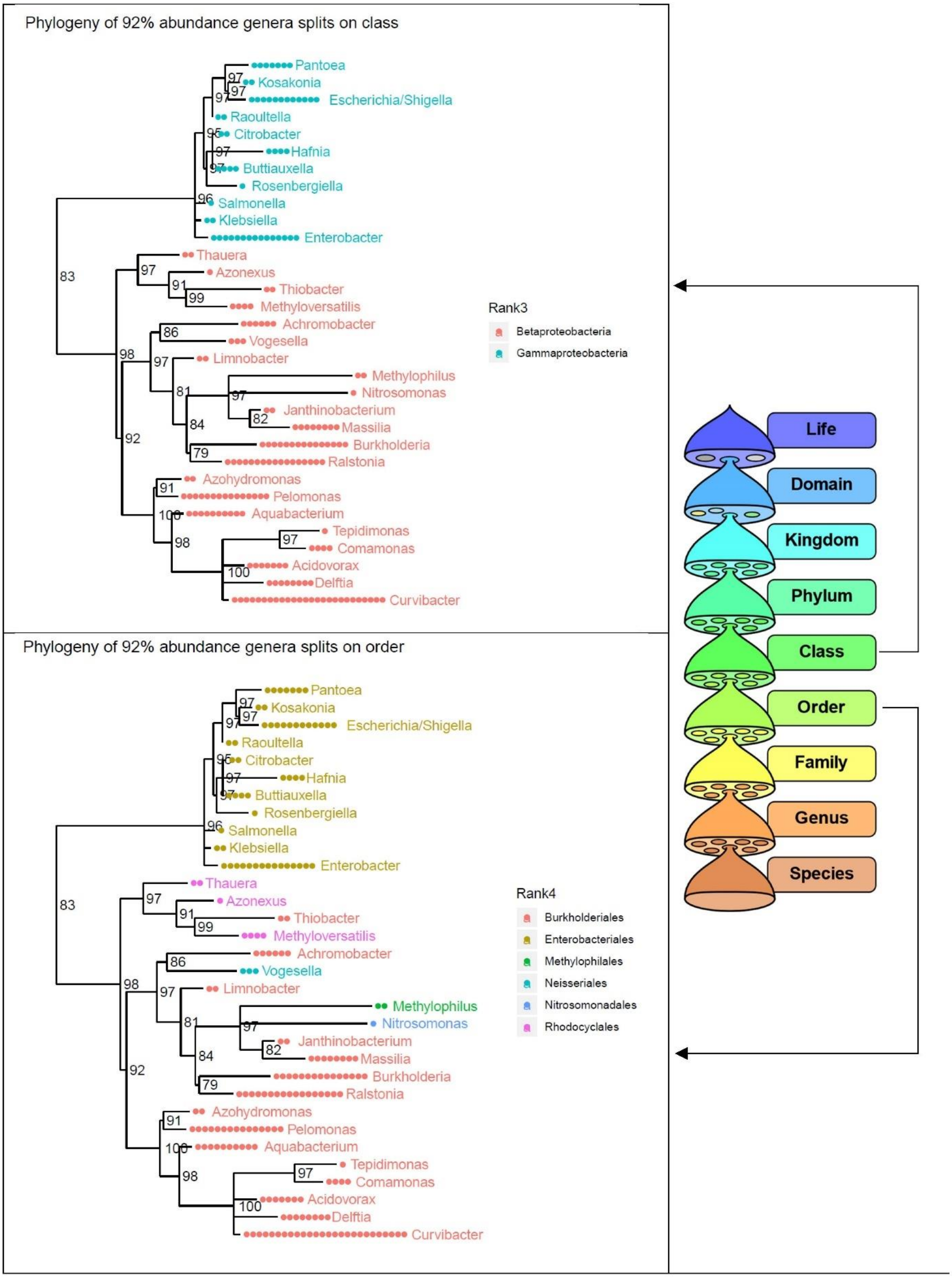

Figure S3. Taxonomic classification and phylogenetic inference. The taxonomic classification and phylogenetic inference were obtained independently. The trees on this figure only include the most abundant ASVs. The classes of the ASVs were perfectly split, and the more abundant orders generally agreed with the phylogenetic inference.

Table S1. Summary of the sample metadata.

Sample_ID	Country	Enzyme_Source	Main_Ingredient	Mean_Temp_C	Month	Product
B1	Benin	Millet	Millet	29	2	Aklui
B10	Benin	Maize	Maize	29	2	Akpan
B11	Benin	Maize	Maize	29	2	Mawe
B2	Benin	Maize	Maize	29	2	Aklui
B3	Benin	Millet	Millet	29	2	Mawe
B4	Benin	Maize	Maize	29	2	Akpan
B5	Benin	Maize	Maize	29	2	Akpan
B6	Benin	Maize	Maize	29	2	Mawe
B7	Benin	Maize	Maize	29	2	Mawe
B8	Benin	Maize	Maize	29	2	Akpan
B9	Benin	Maize	Maize	29	2	Akpan
T1	Tanzania	Millet malt flour	Maize flour	25	1	Togwa
T10	Tanzania	Millet malt flour	Maize flour	25	1	Togwa
T11	Tanzania	Millet malt flour	Maize flour	25	1	Togwa
T12	Tanzania	Millet malt flour	Maize flour	25	1	Togwa
T2	Tanzania	Millet malt flour	Maize flour	25	1	Togwa
T3	Tanzania	Millet malt flour	Maize flour	25	1	Togwa
T4	Tanzania	Millet malt flour	Maize flour	25	1	Togwa
T5	Tanzania	Millet malt flour	Maize flour	25	1	Togwa
T6	Tanzania	Millet malt flour	Maize flour	25	1	Togwa
T7	Tanzania	Millet malt flour	Maize flour	25	1	Togwa
T8	Tanzania	Millet malt flour	Maize flour	25	1	Togwa
T9	Tanzania	Millet malt flour	Maize flour	25	1	Togwa
Z1	Zambia	Wheat flour	Maize meal	16	7	Munkoyo
Z10	Zambia	Cowpea flour	Maize meal	16	7	Munkoyo
Z11	Zambia	*Rhynchosia* root	Maize meal	16	7	Munkoyo
Z12	Zambia	*Rhynchosia* root	Maize meal	16	7	Munkoyo
Z2	Zambia	*Rhynchosia* root	Maize meal	16	7	Munkoyo
Z3	Zambia	Wheat flour	Maize meal	16	7	Munkoyo
Z4	Zambia	*Rhynchosia* root	Maize meal	16	7	Munkoyo
Z5	Zambia	Sweet potato peel	Maize meal	16	7	Munkoyo
Z6	Zambia	*Rhynchosia* root	Maize meal	16	7	Munkoyo
Z7	Zambia	*Rhynchosia* root	Maize meal	16	7	Munkoyo
Z8	Zambia	*Rhynchosia* root	Maize meal	16	7	Munkoyo
Z9	Zambia	Sweet potato peel	Maize meal	16	7	Munkoyo

Table S2. Mann–Whitney U test for different alpha diversity measures. Measures that incorporated the number of ASVs (observed) or an estimate on the number of species (Chao1 and ACE) resulted in significant difference between Benin and the other two countries, whereas measures incorporating number of ASVs and the respective proportions of the ASVs (Shannon and Simpson) resulted in no significant differences between all countries.

Comparison	Observed	Shannon	Simpson	Chao1	ACE
Benin vs. Tanzania	0.009543	0.406048	0.734985	0.001692	0.001526
Benin vs. Zambia	0.001369	0.116551	0.781814	0.000636	0.000796
Tanzania vs. Zambia	0.09364	0.260236	0.506721	0.099877	0.088534

Table S3. Results of PERMANOVA statistical analysis testing differences in amplicon sequence variant richness between samples from all three countries.

	Df	SumsOfSqs	MeanSqs	F.Model	R2	Pr (>F)
Country	2	2.709334	1.354667	3.628282	0.18485	0.001
Residuals	32	11.94762	0.373363	NA	0.81515	NA
Total	34	14.65695	NA	NA	1	NA

Table S4. Results of PERMANOVA statistical analysis testing differences in amplicon sequence variant richness between samples from Benin and Tanzania.

	Df	SumsOfSqs	MeanSqs	F.Model	R2	Pr (>F)
Country	1	0.674268	0.674268	1.634406	0.072209	0.015
Residuals	21	8.663475	0.412546	NA	0.927791	NA
Total	22	9.337743	NA	NA	1	NA

Table S5. Results of PERMANOVA statistical analysis testing differences in amplicon sequence variant richness between samples from Benin and Zambia.

	Df	SumsOfSqs	MeanSqs	F.Model	R2	Pr (>F)
Country	1	1.592925	1.592925	4.581864	0.179106	0.001
Residuals	21	7.30083	0.347659	NA	0.820894	NA
Total	22	8.893755	NA	NA	1	NA

Table S6. Results of PERMANOVA statistical analysis testing differences in amplicon sequence variant richness between samples from Tanzania and Zambia.

	Df	SumsOfSqs	MeanSqs	F.Model	R2	Pr (>F)
Country	1	1.778385	1.778385	4.933148	0.183163	0.001
Residuals	22	7.930933	0.360497	NA	0.816837	NA
Total	23	9.709317	NA	NA	1	NA

Table S7. Zambian samples compared based on the presence of root material. No significant difference was found between samples with added root material and those without one.

	Df	SumsOfSqs	MeanSqs	F.Model	R2	Pr (>F)
Root	1	0.333095	0.333095	0.897866	0.082389	0.593
Residuals	10	3.709854	0.370985	NA	0.917611	NA
Total	11	4.042949	NA	NA	1	NA

In tables S3–S7, Df represents degrees of freedom; SumsOfSqs represents the sum of squares. MeanSqs is the mean square error, calculated as SumsOfSqs/Df. F.Model represents the F test value. R2 represents the explanatory degree of different groups to sample differences, calculated as the ratio of group variance to total variance. Pr(>F) represents p-value, where $p < 0.05$ indicates that there is a statistically significant difference in this grouping level.
